# Augmentation Cystoplasty and Extracellular Matrix Scaffolds: An *Ex Vivo* Comparative Study with Autogenous Detubularised Ileum

**DOI:** 10.1371/journal.pone.0020323

**Published:** 2011-05-25

**Authors:** Niall F. Davis, Rory Mooney, Anthony Callanan, Hugh D. Flood, Tim M. McGloughlin

**Affiliations:** 1 Department of Urology, Mid-Western Regional Hospital, Limerick, Ireland; 2 Centre for Applied Biomedical Engineering Research and MSSI, University of Limerick, Ireland; Universidade de Sao Paulo, Brazil

## Abstract

**Background:**

Augmentation cystoplasty (AC) with autogenous ileum remains the current gold standard surgical treatment for many patients with end-stage bladder disease. However, the presence of mucus-secreting epithelium within the bladder is associated with debilitating long-term complications. Currently, decellularised biological materials derived from porcine extracellular matrix (ECM) are under investigation as potential augmentation scaffolds. Important biomechanical limitations of ECMs are decreased bladder capacity and poor compliance after implantation.

**Methodology/Principal Findings:**

In the present *ex vivo* study a novel concept was investigated where a two-fold increase in ECM scaffold surface-area relative to the resected ileal segment was compared in ovine bladder models after AC. Results showed that bladder capacity increased by 40±4% and 37±11% at 10 mmHg and compliance by 40.4±4% and 39.7±6% (ΔP = 0–10 mmHg) after AC with ileum and porcine urinary bladder matrix (UBM) respectively (p<0.05). Comparative assessment between ileum and UBM demonstrated no significant differences in bladder capacity or compliance increases after AC (p>0.05).

**Conclusions:**

These findings may have important clinical implications as metabolic, infective and malignant complications precipitated by mucus-secreting epithelium are potentially avoided after augmentation with ECM scaffolds.

## Introduction

The primary function of the urinary bladder is to store urine between periods of voluntary voiding. However, a number of pathological conditions such as multiple sclerosis, spinal cord injury, and idiopathic urge incontinence may affect the bladder often resulting in a poorly compliant, high-pressure storage reservoir. After failing conservative and medical therapies patients with these conditions often need surgical intervention with augmentation cystoplasty (AC). AC is the addition of viscoelastic autogenous gastrointestinal tissue to the bladder to increase capacity, improve compliance, or decrease detrusor overactivity. AC has replaced cutaneous urinary diversion as the gold standard surgical treatment in this group of patients because of decreased morbidity and improved quality of life. Long-term disadvantages of AC include recurrent urinary tract infections (UTIs), bladder calculi, metabolic disturbances and a predisposition to malignancy [1].

On account of these complications, researchers have recently attempted to use alternative augmentation strategies by applying tissue-engineered extracellular matrix (ECM) scaffolds in place of autogenous gastrointestinal tissue [2], [3]. ECMs are decellularised, biodegradable membranes usually derived from porcine organs [4]. Furthermore, post-operative inflammatory reactions are avoided with ECMs due to their acellular inert nature [5]. Urinary bladder matrix (UBM) is an ECM that is sourced from the porcine bladder and it induces a host derived regenerative response after surgical implantation [6]. Replacing autogenous ileum with UBM for bladder augmentation purposes may have the advantages of fewer long-term metabolic, infective and malignant complications while negating autogenous ileal resection.

Despite the perceived functional advantages of ECMs compared to autogenous ileum, significant biomechanical limitations occur when ECM is applied in urological settings. Postoperatively, bladder capacity and compliance remain significantly lower than values achieved with more compliant ileal segments [7], [8]. To prevent these biomechanical complications from occurring, it may be necessary to increase the ECM scaffold surface-area, relative to the surface-area of detubularised ileum, if comparable capacity and compliance values are to be achieved after AC [9]. The aim of the present *ex vivo* study was to determine the relative increase in ECM scaffold surface-area required for achieving comparable bladder capacity values with ileum after AC in ovine bladder models.

## Materials and Methods

### 2.1 Ethics statement

All animal tissue was obtained from an abattoir (Gaelic Meats, Patrickswell, Limerick, Ireland) immediately after euthanasia. Ethical approval for *ex vivo* tissue culture was approved by the University of Limerick's ethics approvals process.

### 2.2 Overview of experimental design

Urinary bladders and distal ileum of 17 female lambs, all 7 months old, were sourced from an abattoir (Gaelic Meats, Patrickswell, Limerick, Ireland) immediately after euthanasia. Porcine UBM was received from the McGowan Institute of Regenerative Medicine, University of Pittsburgh, and all other materials were obtained from CABER (Centre for Applied Biomedical Engineering Research, Limerick, Ireland) unless indicated. Four bladders were used for comparative tensile testing purposes with ileum and UBM. One control bladder was used to determine the relative surface-area ratio of UBM to ileum for achieving comparable bladder capacity values after AC. To validate our ‘surface-area’ concept the remaining 12 bladders underwent AC; 6 had AC with detubularised ileum (5×5 cm) and 6 had AC with a larger (8×6.5 cm) UBM scaffold. The primary endpoint of the study was to compare bladder capacity and compliance in both AC groups.

### 2.3 Biomechanical testing

Strips of bladder tissue were resected along the bladder's longitudinal axis and measured 30 mm×9.3 mm ×1.5±0.29 mm in length, width and thickness respectively. Three strips were obtained from each bladder and 12 strips were prepared for tensile testing purposes. Strips of autogenous detubularised ileum were resected along the longitudinal axis of the small bowel and measured 40 mm×9.3 mm ×0.35±0 mm (n = 12). Strips of UBM measured 40 mm ×9.3 mm ×0.20±0 mm and were prepared for comparative analysis (n = 12). Each material was cut into a dog-bone shape (with a steel template) and maintained in tissue transport medium at 37°C (Dulbecco's phosphate buffered saline, Innoprot®, Bizkaia, Spain). Tensile testing was performed by clamping each prepared specimen into a custom fabricated tensile tester with a 50 N load cell (Mecmesin®, Newton House, Spring Copse Business Park, Slinfold, West Sussex, United Kingdom). An incremental strain rate of 1 mm/s was applied to each clamped tensile specimen and the magnitude of the load (N) was measured in Newtons. Extension data and load obtained during the testing period were converted to stress (σ) and strain (ε) values.

### 2.4 Measurement of baseline bladder capacity and compliance

**Figure 1:**
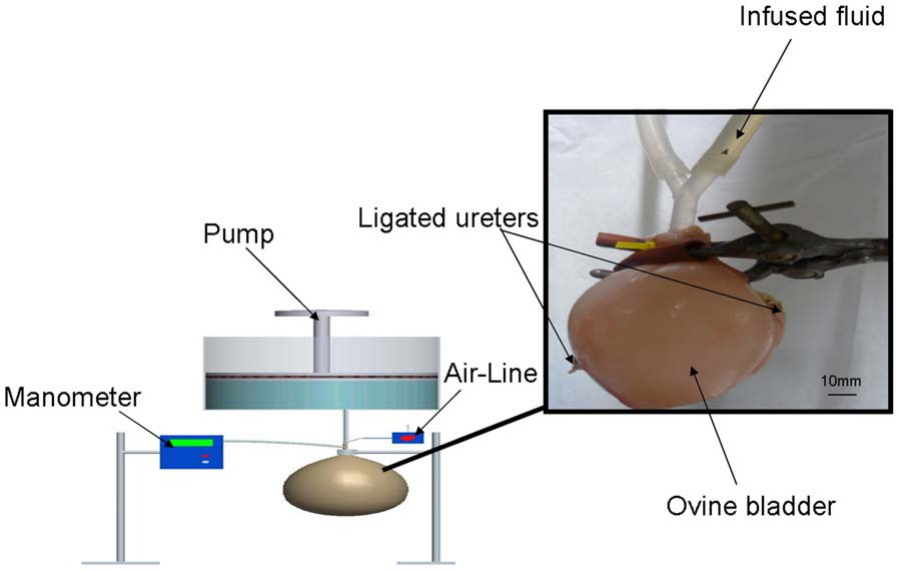
Schematic of ovine bladder connected to pressure/volume rig at 10 mmHg via the urethral orifice. The filling rate into the bladder was 0.5 ml/min and bladder capacity was recorded at 2, 4, 6, 8 and 10 mmHg by measuring the volume (ml) of infused fluid at each pressure (mmHg) measurement.



The urinary bladder and both ureters of each ovine model was identified, mobilised and explanted through dissection techniques. Each explanted bladder was maintained in tissue transport medium (Dulbecco's phosphate buffered saline, Innoprot®, Bizkaia, Spain) and transported to the laboratory on ice immediately after euthanasia. A ‘pressure/ volume’ rig was constructed based on previously described methodology [9]. Each bladder was connected to the rig after emptying all intravesical contents and ligating both ureters. Air was then fed into a fluid-filled vessel at a constant rate via an air line. This resulted in an increase in hydrostatic pressure within the vessel and allowed fluid to flow into each urinary bladder via the urethral orifice at a controlled physiological flow rate of 0.5 ml/min (Fig. 1). Bladder capacity was recorded at 2, 4, 6, 8 and 10 mmHg by measuring the volume (V) of infused fluid at each pressure (P) measurement. Compliance (C) was measured by dividing the volume changes by their relative pressure changes at intravesical pressures of 10 mmHg [10].

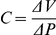
Where: C =  compliance, ΔV =  change in volume, ΔP =  change in pressure.

### 2.5 Preparation of UBM grafts

Preparation of UBM has previously been described [11]. Briefly, the inner urothelial layers are removed by submerging the harvested porcine bladder in normal saline. The outer tunica serosa, tunica muscularis externa, tunica submucosa and muscularis mucosa are then manually delaminated from the bladder. The remaining basement membrane of the tunica epithelialis submucosa and subjacent tunica propria is termed UBM. Finally, the decellularisation process is completed by soaking the UBM scaffold in buffered saline (pH 7.4), placing it in peracetic acid/4% ethanol for 2 hours and rinsing it in sterile buffered saline. Manufacture of the 4-ply sheets is performed by moisture extraction and a vacuum-pump compression technique.

### 2.6 Surface-area ratio of UBM to ileum required for augmentation cystoplasty

To calculate the surface-area ratio of UBM to ileum, baseline bladder capacity was measured in a control bladder at 2 mmHg. AC with 25 cm^2^ of detubularised ileum was performed on the control and percentage increases in capacity were recorded at 2 mmHg (Table 1). Subsequently, AC was performed on the control with 5 UBM grafts that increased in surface-area from 20 cm^2^ to 80 cm^2^. Percentage increases in capacity after AC with UBM (surface-area ranges: 20–80 cm^2^) were recorded at 2 mmHg and compared with ileum. Analysis of the data demonstrated that AC with a 50 cm^2^ UBM scaffold resulted in a 40.7% increase in capacity compared to an increase 40.0% with 25 cm^2^ of detubularised ileum at 2 mmHg (Table 1).

**Table 1:** Calculating the surface-area ratio of UBM to ileum after AC in a control bladder.

	Ileum	UBM 1	UBM 2	UBM 3	UBM 4	UBM 5
**Volume at 2 mmHg (ml)**	***105***	105	105	105	***105***	105
**Dimension of applied graft for AC (cm)**	***5*×*5***	5×4	6×5	7×5.725	***8*×*6.5***	10×8
**Surface area of applied graft (cm^2^)**	***25***	20	30	40	***50***	80
**Surface area ratio of ileum to UBM**	-	1∶0.8	1∶1.2	1∶1.6	***1∶2***	1∶3.2
***Percentage increase in capacity (%)***	***40.0***	23.9	32.3	37.5	***40.7***	50.0

AC, augmentation cystoplasty.

AC was initially performed with ileum (25 cm^2^) and percentage increases in capacity were recorded at 2 mmHg. Subsequently, AC was performed on the control with UBM scaffolds ranging from 20 cm^2^ to 80 cm^2^ and percentage increases were compared at 2 mmHg to ileal values. AC with an 8×6.5 cm (50 cm^2^) UBM scaffold resulted in a 40.7% increase in bladder capacity compared to an increase of 40.0% with a 5×5 cm (25 cm^2^) of detubularised ileum at 2 mmHg. Hence, 50 cm^2^ UBM scaffolds were applied to ovine bladders for comparative purposes with ileum after AC.

### 2.7 Augmentation cystoplasty

A two-fold increase in UBM surface-area (50 cm^2^) relative to autogenous ileum (25 cm^2^) demonstrated comparable increases in bladder capacity after AC with both materials (see methods section 2.6 and table 1). Hence, 50 cm^2^ UBM scaffolds were applied to ovine bladders for comparative purposes with ileum after AC.

#### 2.7.1 Augmentation cystoplasty with ileum

**Figure 2:**
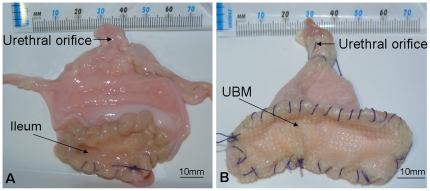
Augmentation cystoplasty with autogenous distal ileum (A) and UBM (B). For the ileocystoplasty the apex of the bladder was bivalved horizontally for 5 cm and detubularised ileum (5×5 cm) was sutured to the bladder (A). For the UBM-cystoplasty the bladder was bivalved 5 cm horizontally along is apex and four-ply UBM (8×6.5 cm) was sutured to the bladder (B).

For the ileocystoplasty technique a 5 cm segment of ileum was resected and detubularised along its antimesenteric border to form a 5×5 cm ileal sheet (n = 6). The bladder was then bivalved horizontally for 5 cm along its apex and the detubularised ileum was sutured to the bladder using a running 3–0 polyglactin suture (Fig. 2a). Water tightness of the anastamosis was confirmed by infusing saline via the urethral orifice.

#### 2.7.2 Augmentation cystoplasty with UBM

Sheets of four-ply UBM scaffolds measuring 8×6.5 cm (50 cm^2^) were pre-hydrated in normal saline for 45 min at 37°C (n = 6). The bladder was then bivalved 5 cm horizontally along is apex. UBM was fixed to the urinary bladder using a running 3–0 polyglactin suture and oriented such that its basement membrane faced the luminal surface of the bladder (Fig. 2b). Water-tightness of the anastomosis was confirmed by injecting saline via the urethral orifice.

### 2.8 Measurement of bladder capacity and compliance in augmented bladders

Bladders augmented with autogenous ileum and UBM were connected to the pressure/volume rig and bladder capacity was measured at 2, 4, 6, 8, and 10 mmHg. Initially, capacity in both augmented groups was compared with baseline values (i.e. capacity prior to AC). Capacity values in both augmented groups were then compared with one another. Subsequently, compliance values at 10 mmHg in both augmented groups was calculated and compared by dividing pressure changes by relative volume changes (ΔV/ΔP).

### 2.9 Statistical analysis

Data was expressed as a mean ± standard deviation. Statistical analysis was performed using a one-factor analysis of variance (ANOVA). Student *t*-tests with unequal variances were performed for pairwise comparisons. Differences were considered significant at p<0.05 (SPSS 16.0 for Windows).

## Results

### 3.1 Biomechanical tensile testing for bladder, ileum and UBM

**Figure 3:**
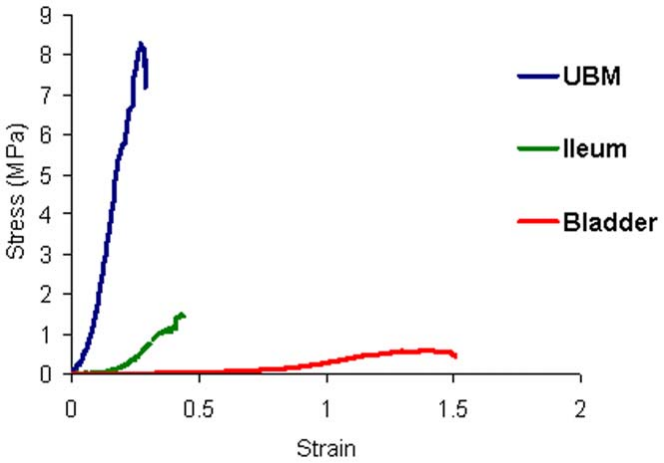
Comparative uni-axial tensile testing between UBM, ileum and bladder tissue. Strips of UBM (40 mm ×9.3 mm ×0.20±0 mm), ileum (40 mm ×9.3 mm ×0.35±0 mm) and bladder tissue (30 mm ×9.3 mm ×1.5±0.29 mm) were loaded into the tensile tester and an incremental extension rate of 1 mm/s was applied. Extension data and load obtained during the testing period were converted to stress (σ) and strain (ε) values for comparative analysis**.**

Stress-strain curves between bladder tissue, ileal tissue and UBM were constructed based on average data as illustrated in figure 3. Strain to failure occurred at 26.2±6.1% in UBM, at 42.2±5.2% in ileum and at 128.3±10% in bladder tissue (p<0.01). Linear stiffness ‘E’ at 30% strain measured 0.066±0.03 MPa for bladder tissue compared to 0.67±0.32 MPa and 16.4±2.51 MPa for ileum and UBM respectively (p<0.01). Table 2 compares the mechanical characteristics of all 3 materials assessed in the study.

**Table 2:** Comparative assessment of uni-axial tensile testing results between UBM, ileum and bladder tissue (*p<0.001).

Parameters	UBM	Ileum	Bladder
Failure Strength (MPa)*	8.23±0.776	1.81±0.47	0.612±0.14
Failure Strain*	26.2±6.1%	42.2±5.2%	128.3±10.0%
Linear Stiffness ‘E’ at 30% strain (MPa)*	16.4± 2.51	0.67±0.32	0.066±0.034
Failure Load (N)*	15.34±1.49	5.16±1.15	8.53±1.87
Failure Extension (mm)*	10.77±2.55	17.78±2.46	44.16±5.13

### 3.2 Augmentation cystoplasty with ileum

**Figure 4:**
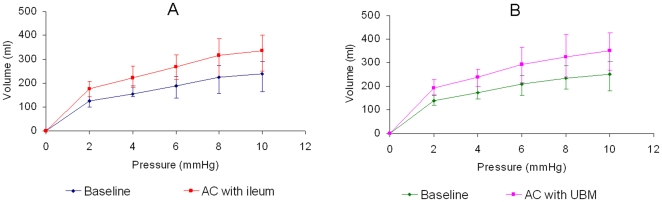
Comparative illustration of bladder capacity (ml) at baseline and after AC with ileum (A) and UBM (B). AC, augmentation cystoplasty.

Baseline and ileocystoplasty capacity values are summarised in Table 3. Baseline capacity values ranged from 125±17 ml at 2 mmHg to 240±45 ml at 10 mmHg and ileocystoplasty values ranged from 175±25 ml at 2 mmHg to 336±56 ml at 10 mmHg. Ovine bladders that underwent AC with autogenous ileum demonstrated a significant increase in bladder capacity at 2, 4, 6, 8 and 10 mmHg (Fig. 4a, p<0.01). Volumetric increases ranged from 51±9 ml at 2 mmHg to 96±13 ml at 10 mmHg. Bladder compliance measured at 10 mmHg was also significantly increased after AC with ileum (40.4±4%, p<0.01). Compliance values ranged from 24±5 ml/mmHg at baseline (ΔP = 0–10 mmHg) and increased to 34±6 ml/mmHg (ΔP = 0–10 mmHg) post AC with ileum (Table 4).

**Table 3:** Bladder capacity characteristics before and after AC with ileum (5×5 cm) at increasing pressures.

Pressure (mmHg)	2	4	6	8	10
**Pre-AC with ileum (ml)**	125±17	155±25	188±34	225±42	240±45
**Post-AC with ileum (ml)**	175±25	221±35	269±44	316±57	336±56
Volumetric increase (ml)*	51±9	66±13	82±12	92±15	96±13
Percentage increase (%)*	40±4	43±7	44±7	41±4	40±4

AC, augmentation cystoplasty.

Volumetric increases after ileocystoplasty ranged from 51±9 ml at 2 mmHg to 96±13 ml at 10 mmHg (*p<0.01, n = 6).

**Table 4:** Comparative assessment of compliance values in both groups before and after AC.

Bladder Compliance (ml/mmHg)	Ileum	UBM	‘p’ value
**Pre-AC (ml/mmHg)**	24±5	25±5	-
**Post-AC (ml/mmHg)**	34±6	35±7	<0.02*
**Percentage Increase (%) (p†)**	40.4±4	39.7±6	(>0.8†)

AC, augmentation cystoplasty.

Compliance values were obtained by dividing volume changes by their relative pressure changes at 10 mmHg. Values were also compared between both groups after AC.

*Comparing baseline compliance values with values post AC in both groups.

† Comparing percentage increases in compliance between ileum and UBM.

### 3.3 Augmentation cystoplasty with UBM

Baseline and UBM-cystoplasty capacity values are summarised in Table 5. Baseline bladder capacities in the group undergoing AC with UBM were greater than the ileocsystoplasty group; however these differences were not statistically significant (p>0.1). Baseline values in the second group ranged from 139±14 ml at 2 mmHg to 266±52 ml at 10 mmHg. Capacity values were significantly increased after augmentation with UBM and volumetric increases ranged from 52±16 ml at 2 mmHg to 100±25 ml at 10 mmHg (Fig. 4b, p<0.05). Compliance at ΔP of 0–10 mmHg was also increased after AC with UBM (39.7±6%) in comparison to baseline. Compliance values ranged from 25±5 ml/mmHg (ΔP = 0–10 mmHg) to 35±7 ml/mmHg (ΔP = 0–10 mmHg) post AC with UBM (Table 5, p<0.02).

**Table 5:** Bladder capacity characteristics at before and after AC with UBM (8×6.5 cm) at increasing pressures.

Pressure (mmHg)	2	4	6	8	10
**Pre-AC with UBM (ml)**	140±14	174±22	210±35	234±48	266±52
**Post-AC with UBM (ml)**	192±25	239±40	292±65	324±79	352±74
Volumetric increase (ml)*	52±16	65±26	83±34	90±36	100±25
Percentage increase (%)*	37±11	37±14	39±13	38±12	39±6

AC, augmentation cystoplasty.

Volumetric increases after AC with UBM ranged from 52±16 ml at 2 mmHg to 100±25 ml at 10 mmHg. Percentage increases in capacity ranged from 37±11% at 2 mmHg to 39±6% at 10 mmHg (*p<0.02, n = 6).

### 3.4 Comparison of bladder capacity and compliance in both augmented groups

**Figure 5:**
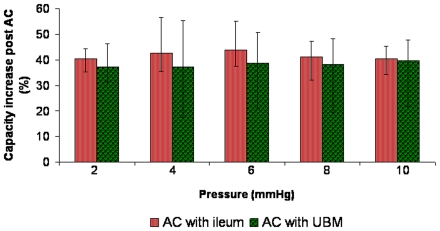
Comparative illustration of percentage increases in bladder capacity after AC with ileum and UBM. Bladder capacity increases were not statistically significant at 2, 4, 6, 8 and 10 mmHg when AC with ileum (5×5 cm) was compared with UBM (8×6.5 cm), (p>0.5). AC, augmentation cystoplasty.

Comparisons of bladder capacity characteristics between both groups that underwent AC are summarised in Table 6. Ovine models undergoing AC with autogenous ileum showed a volumetric increase of 96±13 ml at 10 mmHg whereas models that underwent AC with UBM demonstrated an increase of 100±25 ml (p>0.5). Percentage increases in bladder capacity were greater in ovine models that underwent AC with ileum compared to AC with UBM and ranged from 3±12% at 2 mmHg to 1±5% at 10 mmHg, however these values were not statistically significant (Fig. 5, p>0.5). Compliance increases at 10 mmHg were also greater, but not statistically significant, in ovine models undergoing AC with ileum compared to AC with UBM (40.4±4% versus 39.7±6%); (Table 4, p>0.8).

**Table 6:** Comparative assessment of percentage increases in bladder capacity at increasing pressures after AC was performed with ileum and UBM.

Pressure (mmHg)	2	4	6	8	10
**AC with ileum (% increase)**	40±4	43±7	44±7	41±4	40±4
**AC with UBM (% increase)**	37±11	37±14	39±13	38±12	39±6
Percentage Difference (%)*	3±12	6±17	5±14	3±11	1±5

AC, augmentation cystoplasty.

Percentage increases in capacity were greater in ovine models that underwent AC with ileum compared to UBM and ranged from 3±12% at 2 mmHg to 1±5% at 10 mmHg. These values were not statistically significant (*p>0.5).

## Discussion

Bladder augmentation with tissue-engineered ECM scaffolds is challenged by the urinary bladder's unique biomechanical properties. An ideal augmentation scaffold should accommodate large changes in intravesical volume while maintaining almost constant pressure values. Autogenous ileum possesses relatively similar biomechanical properties and has therefore remained the gold standard replacement material since initially described almost 100 years ago [12]. In the present *ex vivo* study we compared AC using UBM with detubularised ileum in an ovine model. Important findings were that AC with UBM resulted in a significant increase in bladder capacity and compliance compared to baseline values. Furthermore, percentage increases in capacity and compliance were comparable in both experimental groups when a two-fold increase in ECM scaffold surface-area (50 cm^2^) relative to the resected ileal segment (25 cm^2^) was applied.

To the authors' knowledge this is the first study comparing UBM with gold-standard autogenous ileum specifically for the purpose of bladder augmentation. A two-fold increase in ECM surface-area was selected based on comparative analysis of volume (ml)/ surface-area (cm^2^) ratios after AC was performed with ileum and 5 UBM scaffolds (varying in surface area from 20–80 cm^2^) on a control bladder model. Analysis of this preliminary data demonstrated that AC with a 50 cm^2^ UBM scaffold resulted in a 40.7% increase in capacity compared to an increase of 40.0% with 25 cm^2^ (5×5 cm) of detubularised ileum. Admittedly, human patients often require 15 cm of autogenous ileum to significantly increase bladder capacity after AC; however ovine bladder capacities are lower than human capacities by a ratio of 3∶1 [13]. Hence, 5 cm ileal segments were applied to accommodate this ratio. After AC percentage increases in bladder capacity were comparable between UBM (39.7±6% at 10 mmHg) and ileum (40±4% at 10 mmHg). These findings may have important clinical implications as metabolic, infective and malignant complications precipitated by mucus-secreting epithelium are potentially avoided after augmentation with UBM.

Previously, ECMs applied for bladder reconstruction have been regular quadrilateral with 4 equal sides (e.g. 5×5 cm) [7], [14]. Importantly, rectangular dimensions (8×6.5 cm) were selected in the current study based on recent investigations by Parekh *et al.* where deformations of the rat bladder were assessed *ex vivo* during the filling process [15]. In their study it was demonstrated that bladder tissue remains stiffer in the circumferential direction compared to the longitudinal direction in passive and inactive states (2.3 and 1.9 peak stretch values longitudinally and circumferentially respectively). These findings infer a peak stretch ratio of 1∶0.8 in favour of the longitudinal direction as the bladder fills. To comply with this longitudinal strain-path to filling, rectangular UBM scaffolds were constructed and implanted in favour of the longitudinal direction (1∶0.8). In the future, we hope to further refine our implantation technique by characterising the major axis of each four-ply UBM scaffold prior to AC This may be achievable by resecting a segment from each multilaminate UBM scaffold prior to surgical implantation and performing bi-axial tensile testing on the resected portion.

It is well established that ECMs possess a number of noteworthy advantages for urological tissue-engineering purposes. Surface-area increases with ECMs are easily achieved due to their xenogenic, readily available nature in comparison to the often restricted availability of autogenous gastrointestinal tissue [13], [16]. After application to the genitourinary tract ECM stimulates a host-derived tissue remodeling response while undergoing simultaneous degradation processes [6]. During the remodeling process activated growth factors are released from their proteoglycans to promote tissue neovascularisation and host-cell deposition. In addition, studies have demonstrated that ECMs are rapidly degraded after implantation into the genitourinary tract with 90% of the scaffold replaced by host tissue within 28 days of implantation [17], [18]. Inevitably, the process may be prolonged (e.g.90–120 days) when multilaminate ECM scaffolds are implanted. After the degradation process ECM scaffolds enter the blood stream and are eventually excreted in the host's urine as demonstrated in one study where the excretion rate of ^14^C labeled ECM was measured in a canine model. Results from this study demonstrated 95% of the scaffold's degradation products were found in the host's urine during the follow up period [18].

However, it is noteworthy that ECMs demonstrate a significant decrease in mechanical strength after implantation due to a temporal imbalance between the rate of scaffold degradation and host cell deposition [5]. This temporal mismatch shifts in favour of the degradation rate and greatly contributes to postoperative complications such as poor compliance, decreased bladder capacity and significantly impaired renal function. Complications of this nature may persist unabated until the remodelling response has been completed after 90–120 days [7], [19]. Preliminary studies in our laboratory have demonstrated that an increase in ECM surface-area relative to its intended bladder defect may prevent these complications by increasing the storage reservoir of the bladder [9]. Intuitively, an increase in the bladder's storage reserve provides the potential for increased capacity at physiological bladder pressures.

Our initial mechanical testing was performed based on previous described methodology and our results clearly demonstrate the relative stiffness of UBM (16.4±2.51% strain to failure) compared to autogenous ileum and bladder tissue (0.67±0.32% and 0.066±0.034% strain to failure respectively, p<0.01) [20], [21], [22]. To compensate for UBM's stiff nature we sought to develop our ‘surface-area’ concept and assess its clinical potential specifically for bladder augmentation purposes. Typically, AC involves an incision along the apex of the bladder and augmentation of the incised surfaces with a suitable biomaterial or scaffold. Recently, investigators have focussed on more appealing, complicated and, perhaps, rewarding applications for ECMs in reconstructive urology. In the field of regenerative medicine, partial or complete bladder reconstruction with ECM has gained widespread interest among many research groups. Arguably, the strong focus on complete organ reconstruction has meant that ECMs have been overlooked as effective candidates for bladder augmentation. A paucity of literature is available on AC with ECMs, however one recent study by Wang *et al*. assessed AC with autogenous ileum and small intestinal submucosa (SIS) in a porcine model [19]. Findings from their study demonstrated greater capacity increases in bladder's augmented with ileum (211 ml) compared to SIS (144.3 ml) after a 6 week experimental time period. Interestingly, the surface-area of ileal and ECM scaffolds applied for AC were equivalent. It is likely that comparable bladder capacity increases between both groups may have been achieved if increases in SIS scaffold surface-area relative to detubularised ileum were applied intraoperatively.

Currently, a variety of biomaterials are commercially available for regenerative and reconstructive purposes across numerous surgical subspecialties. UBM was selected for AC in this study based on notable biomechanical and biological advantages over other ECM scaffolds. Native bladder tissue is mildly anisotropic in response to large intravesical volume increases and remains stiffer along its circumferential axis compared to its longitudinal axis [15]. Recent studies have demonstrated that this *in vivo* stretch response is retained by UBM after the decellularisation process [23]. Conversely, decellularised porcine small intestinal submucosa (SIS) is strongly anisotropic as spirally arranged collagen fibers stretch and constrict along their longitudinal axis in a manner similar to gastrointestinal peristalsis [24]. Interestingly, UBM uniquely retains an intact basement membrane [11] and this advantageous feature can partially contribute to the prevention of urine infiltrating deeper into tissues while supporting growth and differentiation of multilayered urothelial cells *in vivo* [25]. Finally, *in vitro* studies have recently demonstrated that UBM's biocompatibily is significantly better than SIS's when culturing human urothelial cells on its luminal surface prior to *in vivo* implantation [26].

The present study is not without limitations and our results must be viewed with caution. Due to its *ex vivo* nature long-term clinical sequelae of AC with larger UBM scaffolds were not addressed. An *in vivo* study with appropriate pre- and postoperative urodynamic studies would be required to accurately assess functional bladder capacity and effective host-cell infiltration of UBM during the follow-up period. Based on the results of the present study we are currently in the process of designing an *in vivo* study of this nature. Importantly, recent reports describing residual porcine DNA on ECM's after their preparation processes have inevitably led to concerns over *in vivo* biocompatibility [27]. Standardised and thorough decellularistion techniques need to be adopted by researchers to promote host biocompatibility after implantation of the scaffold [16]. It can also be argued that fatigue properties of *ex vivo* bladder and ileal tissue may have biased our capacity results in favour of UBM after AC. However, a number of recent studies have demonstrated that bladder tissue retains its visocoelastic properties and produces reliable results when investigating capacity for up to 48 hours post mortem. To eliminate this potential bias all investigations in the present study were carried out within 6 hours of euthanasia.

ECM scaffolds possess important biological advantages when compared to mucus-secreting ileal tissue for bladder augmentation; however their biomechanical limitations need to be addressed and resolved prior to clinical implementation. In this study we investigated a novel concept where a two-fold increase in ECM scaffold surface-area relative to the resected ileal segment was applied for bladder augmentation in ovine bladder models. Our results demonstrated that this surface-area increase leads to comparable bladder capacity increases after AC. Knowledge obtained from this study provides evidence that ECM scaffolds are an attractive alternative to autogenous gastrointestinal tissue for bladder augmentation purposes.
